# Supplementary material for: SLC1A3 contributes to L‐asparaginase resistance in solid tumors
Source: EMBO J. 2019 Sep 16;38(21):e102147. doi: 10.15252/embj.2019102147 (PMC6826201; doi:10.15252/embj.2019102147)
Supplement: Supplementary file 1 — Expanded View Figures PDF [file EMBJ-38-e102147-s001.pdf]

Expanded View Figures

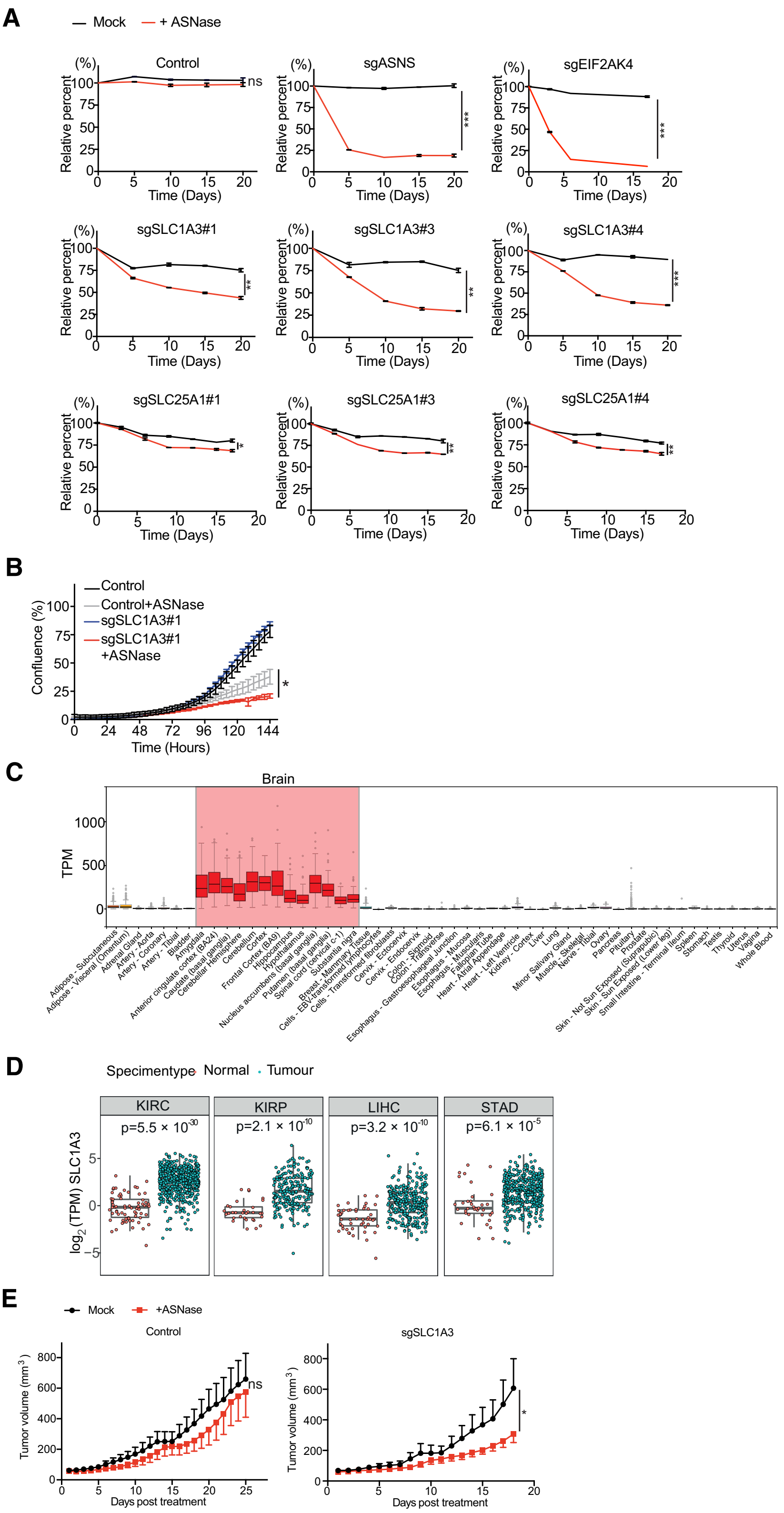

Figure EV1. Validation of hits from the genome-wide CRISPR-Cas9 screen in PC3 cells.

A PC3 cells were transduced with individual sgRNA lentiviral vectors as indicated and subjected to competitive cell proliferation assays under mock or ASNase (0.3 U/ml) conditions. #1, #3, and #4 represent different sgRNAs and $n = 2$ for each condition.

B PC3 cells were transduced with control (sgNon-targeting) or sgSLC1A3 and subjected to IncuCyte cell proliferation assays with or without ASNase (0.3 U/ml). #1 indicates a sgRNA targeting SLC1A3 and $n = 3$ for each condition.

C SLC1A3 expression analysis in normal tissues from GTEX portal (gtexportal.org/home/gene/SLC1A3). Red columns indicate high SLC1A3 expression in most brain tissues. Expression values are shown in TPM (transcripts Per Million), calculated from a gene model with isoforms collapsed to a single gene. No other normalization steps have been applied. Box plots are shown as median and 25th and 75th percentiles; points are displayed as outliers if they are above or below 1.5 times the interquartile range.

D TCGA tumor database analysis of SLC1A3 expression in healthy tissues and primary tumors for kidney renal clear cell carcinoma (KIRC), kidney renal papillary cell carcinoma (KIRP), liver hepatocellular carcinoma (LIHC), and stomach adenocarcinoma (STAD). Expression data from tumor and normal tissue samples were downloaded for every project available at ICGC data portal (http://dcc.icgc.org; release 27). For consistency, only expression data from pipeline "RNASeqV2_RSEM_genes" were considered. The downloaded normalized expression data were scaled to TPM (transcripts per million reads) and log2 transformed. Only projects with more than 10 normal samples were considered (each dot represents one sample). All analyses were done using R-language. The statistical comparison between normal and tumor samples was done using a non-parametric Wilcoxon sum rank test followed by Bonferroni correction. Box plots are shown as median and 25th and 75th percentiles; points are displayed as outliers if they are above or below 1.5 times the interquartile range.

E Control (sgNon-targeting#1) and SLC1A3 knockout (sgSLC1A3#4-1) PC3 cell lines were subcutaneously injected into Balb/c nude mice (cAnN/Rj; $n = 8$ per group). Once tumor volumes reached 50 mm^3, mice were treated with mock (saline) or ASNase (60 U per day). Data were presented as mean ± SEM.

Data information: Results were presented as mean ± SD, unless otherwise stated. The *P*-value was calculated by two-tailed unpaired *t*-test from Prism7. $*P < 0.05$, $**P < 0.01$, $***P < 0.001$.

© 2019 The Authors

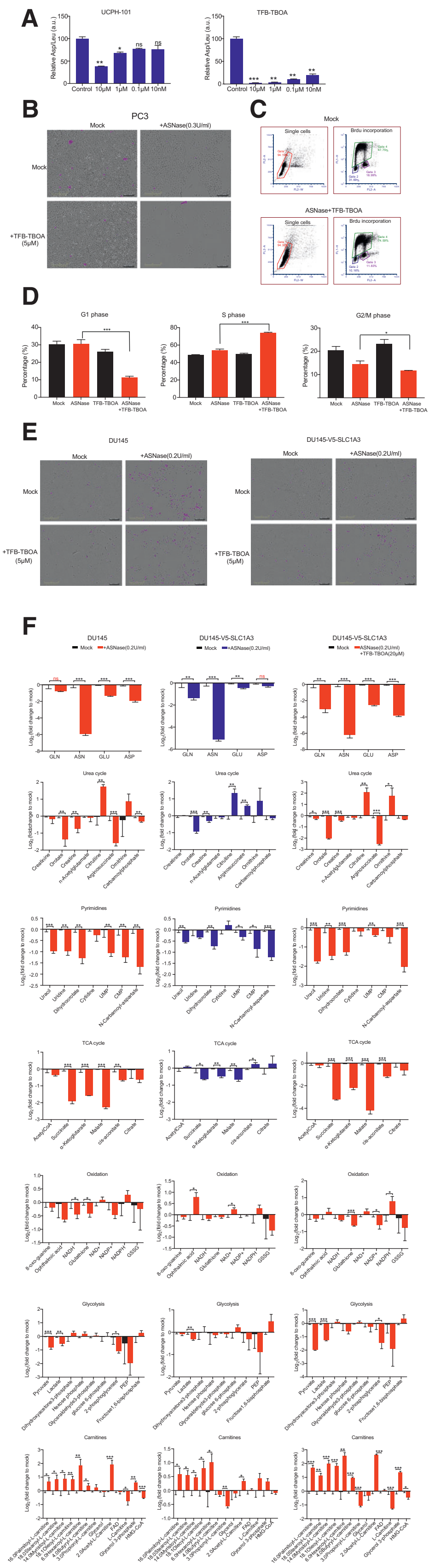

Figure EV2. Combination of ASNase and SLC1A3 inhibition impairs cancer cell proliferation.

- A PC3 cells were incubated with two SLC1A3 inhibitors (UCPH-101 and TFB-TBOA) at indicated concentrations and subjected to an aspartate uptake assay (*n* = 2). Leucine uptake level was used for normalization.
- B PC3 cells were treated with ASNase (0.3 U/ml) and TFB-TBOA (5 μM) as indicated and images were taken after 4 days. The scale bar indicates 300 μm and cells highlighted in pink present apoptotic cells.
- C PC3 cells were treated with mock or combination of ASNase (0.3 U/ml) and TFB-TBOA (5 μM) for 9 days and subjected to flow cytometry measurements of cell cycle using BrdU labeling.
- D Flow cytometry cell cycle distribution analysis of PC3 cells under indicated conditions for 9 days by BrdU labeling. ASNase (0.3 U/ml) and TFB-TBOA (5 μM).
- E Representative images of apoptosis in DU145 and DU145-V5-SLC1A3 cells treated with ASNase (0.2 U/ml) and TFB-TBOA (5 μM) as indicated, and subjected to IncuCyte analysis for apoptotic cells (highlighted in pink). The scale bar indicates 300 μm.
- F DU145 and DU145-V5-SLC1A3 cells were treated with ASNase (0.2 U/ml) and TFB-TBOA (20 μM) for 3 days as indicated, and collected for metabolome analysis by LC-MS to determine the relative levels of amino acids and metabolites involved in urea cycle, pyrimidine synthesis, TCA cycle, oxidation, glycolysis, and carnitines. GLN, glutamine; ASN, asparagine; GLU, glutamate; ASP, aspartate; TCA cycle, tricarboxylic acid cycle; UMP, uridine monophosphate; CMP, cytidine monophosphate; PEP, phosphoenolpyruvate; NADH, nicotinamide adenine dinucleotide (reduced form); NAD^+, nicotinamide adenine dinucleotide (oxidized form); NADPH, nicotinamide adenine dinucleotide phosphate (reduced form); $NADP^+$, nicotinamide adenine dinucleotide phosphate (oxidized form); GSSG, glutathione disulfide; HMG-CoA, 3-hydroxy-3-methylglutaryl-CoA; FAD, flavin adenine dinucleotide.

Data information: Results were calculated based on three independent replicates, unless otherwise stated, and presented as mean ± SD. The *P*-value was calculated by two-tailed unpaired *t*-test from Prism7. *$P < 0.05$, **$P < 0.01$, ***$P < 0.001$.

© 2019 The Authors

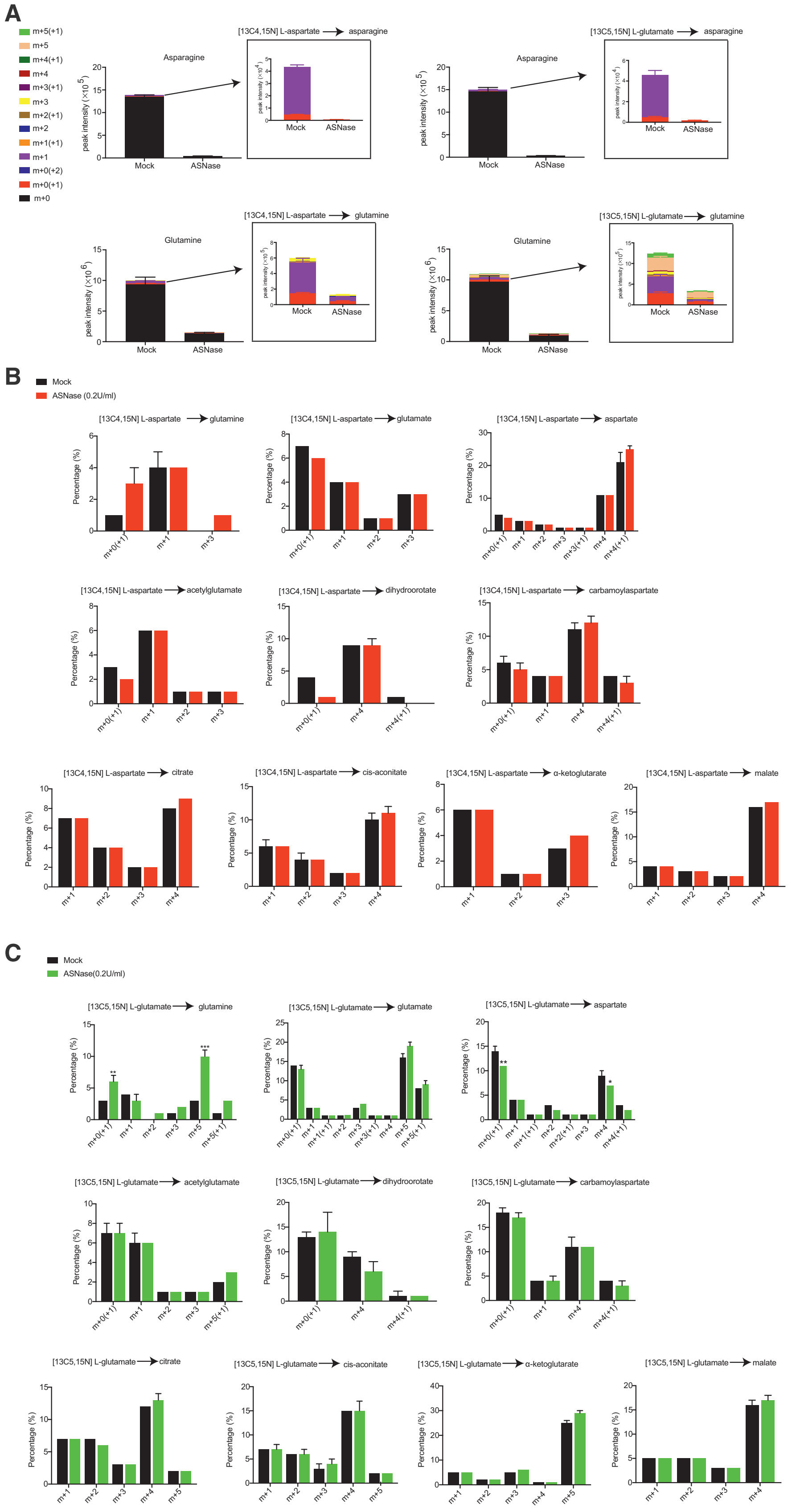

Figure EV3. Isotopic tracing of aspartate and glutamate in DU145-V5-SLC1A3 cells.

DU145-V5-SLC1A3 cells were pretreated with ASNase (0.2 U/ml) for 48 h and then supplemented with [$^{13}C_4$,^{15}N] L-aspartate (1.5 mM) and unlabeled glutamate (1.5 mM), or [$^{13}C_5$,^{15}N] L-glutamate (1.5 mM) and unlabeled aspartate (1.5 mM) for 8 h. Subsequently, cells were harvested for LC-MS analysis.

A Mass isotopologue analysis of ^{13}C and ^{15}N incorporation into asparagine and glutamine in DU145-V5-SLC1A3 cells cultured as indicated conditions.

B Mass isotopologue analysis of ^{13}C and ^{15}N incorporation into glutamine, glutamate, and aspartate and metabolites from TCA cycle, urea cycle, and nucleotide synthesis in DU145-V5-SLC1A3 cells cultured with [$^{13}C_4$,^{15}N] L-aspartate and unlabeled glutamate.

C Mass isotopologue analysis of ^{13}C and ^{15}N incorporation into glutamine, glutamate, and aspartate and metabolites from TCA cycle, urea cycle, and nucleotide synthesis in DU145-V5-SLC1A3 cells cultured with [$^{13}C_5$,^{15}N] L-glutamate and unlabeled aspartate.

Data information: Data shown in (B–C) were calculated as labeled fraction divided by total peak area (including unlabeled fraction), and results were presented as mean ± SD (n = 3). Number before brackets accounts for ^{13}C incorporation and number in brackets accounts for ^{15}N incorporation. The P-value was calculated by two-tailed unpaired t-test from Prism7. *$P < 0.05$, **$P < 0.01$, ***$P < 0.001$.

© 2019 The Authors

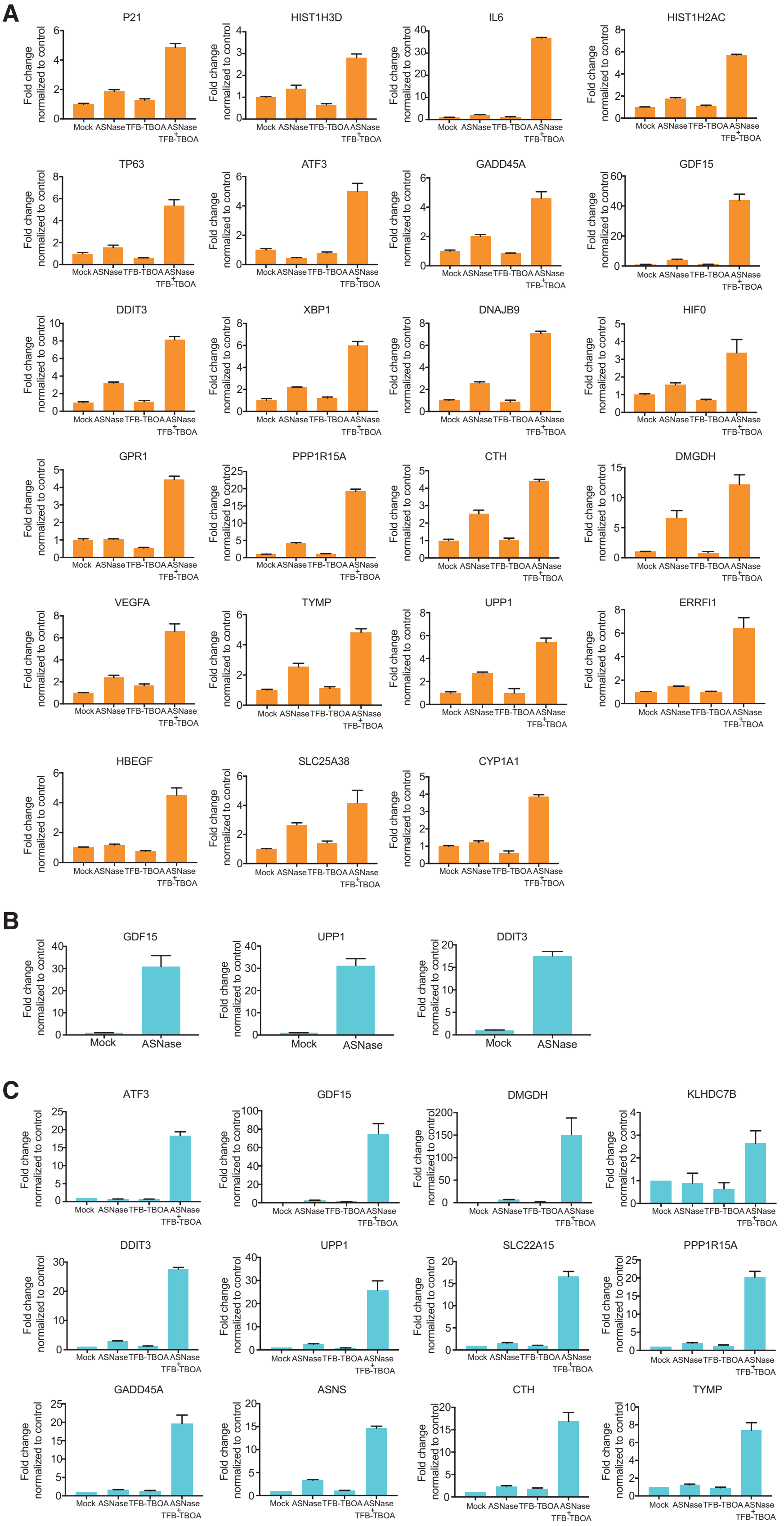

Figure EV4. RT–qPCR validation of differential expressed genes in PC3, DU145, and DU145-V5-SLC1A3 Cells from Fig 4.

A–C RT–qPCR validation based on transcriptome analysis in PC3 (A), DU145 (B), and DU145-V5-SLC1A3 cells (C).

Data information: Results were calculated based on three independent replicates, and presented as mean ± SD.

© 2019 The Authors

Figure EV5. SLC1A3 expression promotes ASNase resistance and tumor progression *in vivo*.

A Glutamine levels in serum, mammary fat pad tissues, and growing tumors derived from SUM159PT human breast cancer cells with or without ASNase treatment (60 U per day) for consecutive 5 days. Essential amino acids were used for the raw data normalization. Results were presented as mean ± SEM (n = 3).

B Left: quantification of aspartate uptake in parental 4T1 and 4T1-V5-SLC1A3 cells (n = 2). Leucine uptake was used for normalization. The P-value was calculated by one-tailed unpaired t-test in Prism7. Right: IncuCyte cell proliferation curves for parental 4T1 and 4T1-V5-SLC1A3 cells with or without ASNase (0.2 U/ml) for 10 days (n = 3).

C Tumor volumes resulting from orthotopic implantation of parental 4T1 and 4T1-V5-SLC1A3 cells at day 12 (n = 13 mice per group, except for 4T1 + ASNase, n = 12). Results were presented as mean ± SEM.

D Days for orthotopically injected parental 4T1 and 4T1-V5-SLC1A3 cells to reach 450–550 mm^3. Results were presented as mean ± SEM (n = 13).

E Following the mastectomy, mice were daily challenged for breathing test and sacrificed due to breathing problems. The survival rate for 4T1- and 4T1-V5-SLC1A3-injected mice was scored in Prism7.

F Left: quantification of aspartate uptake in MDA-MB-231 and MDA-MB-231-V5-SLC1A3 cells (n = 2). Leucine uptake was used for normalization. Right: IncuCyte cell proliferation measurements for MDA-MB-231 and MDA-MB-231-V5-SLC1A3 cells with or without ASNase (1 U/ml) for 8 days (n = 3).

Data information: Results were calculated based on three replicates and presented as mean ± SD (unless otherwise stated). The P-value was calculated by two-tailed unpaired t-test except for (B) in Prism7. $^*P < 0.05$, $^{**}P < 0.01$, $^{***}P < 0.001$.

© 2019 The Authors

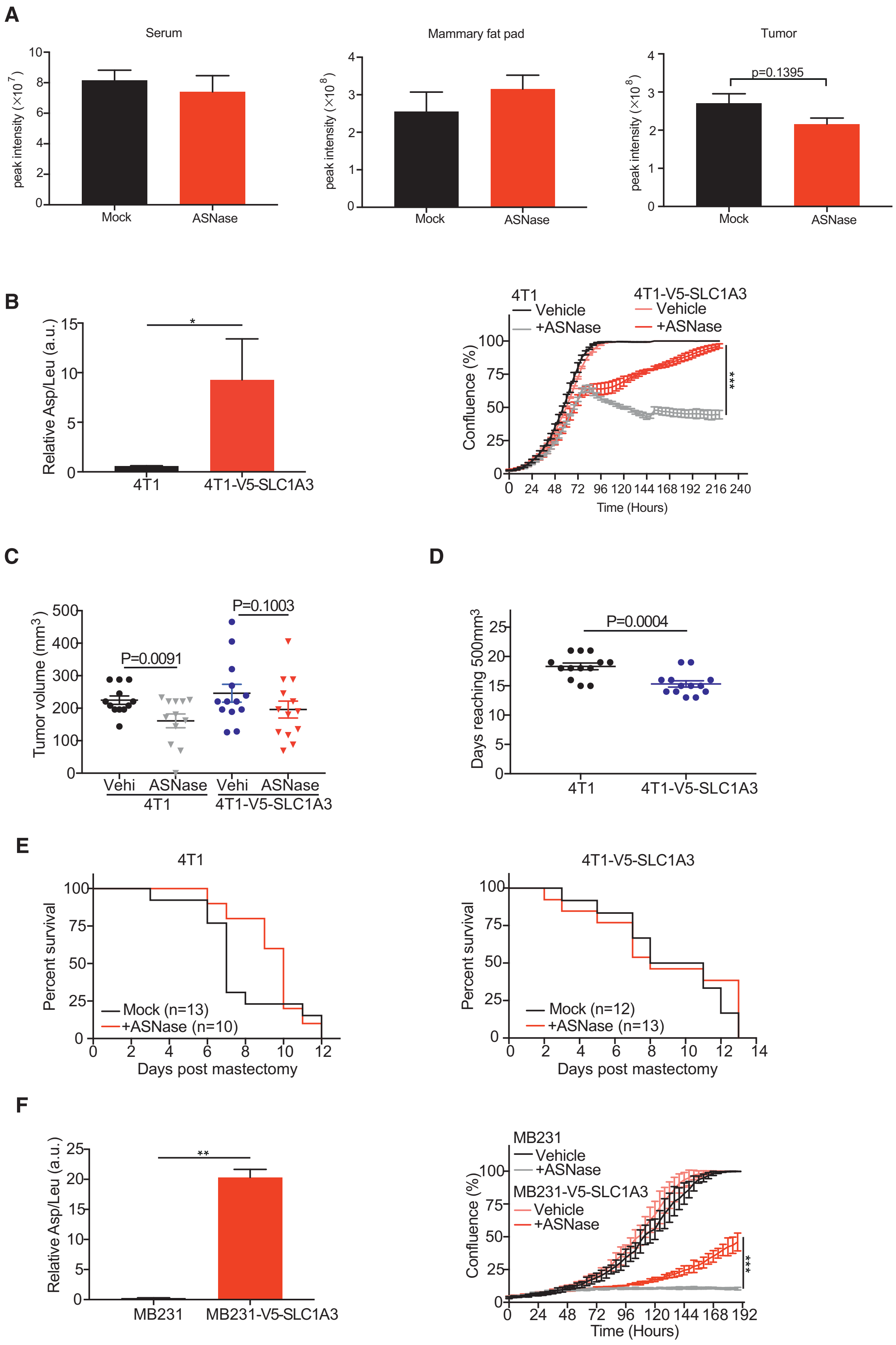

Figure EV5.

© 2019 The Authors
