# Supplementary material for: SLC1A3 contributes to L‐asparaginase resistance in solid tumors
Source: EMBO J. 2019 Sep 16;38(21):e102147. doi: 10.15252/embj.2019102147 (PMC6826201; doi:10.15252/embj.2019102147)
Supplement: Supplementary file 2 — Table EV1 [file EMBJ-38-e102147-s002.docx]

Table EV1: Primers used for sgRNAs and qRT-PCR

| Primers | Sequences (5’-3’) |
| --- | --- |
| sgNon-targeting Forward | GGAGAAATTCTAATGCGGATGCTGA |
| sgNon-targeting Reverse | TTCCGGATACGTTGGAATCCAGT |
| sgASNS Forward | CACCGTTGTCATAGAGGGCGTGCAG |
| sgASNS Reverse | AAACCTGCACGCCCTCTATGACAAC |
| sgEIF2AK4 Forward | CACCGACTGGCCAAGAAACACTGTG |
| sgEIF2AK4 Reverse | AAACCACAGTGTTTCTTGGCCAGTC |
| sgSLC1A3#1 Forward | CACCGACCATACAGAATGAGCTACC |
| sgSLC1A3#1 Reverse | AAACGGTAGCTCATTCTGTATGGTC |
| sgSLC1A3#2 Forward | CACCGACCCTCCAATAAAAACCCAA |
| sgSLC1A3#2 Reverse | AAACTTGGGTTTTTATTGGAGGGTC |
| sgSLC1A3#3 Forward | CACCGGACTCTTACCCGAATCACAG |
| sgSLC1A3#3 Reverse | AAACCTGTGATTCGGGTAAGAGTCC |
| sgSLC1A3#4 Forward | CACCGTCAGGAAGAGAATACCCACG |
| sgSLC1A3#4 Reverse | AAACCGTGGGTATTCTCTTCCTGAC |
| sgSLC1A3#J23 Forward | CACCGTACACCGTGACTGTCATTGT |
| sgSLC1A3#J23 Reverse | AAACACAATGACAGTCACGGTGTAC |
| sgSLC25A1#1 Forward | CACCGCTGCGTCTTCACGTACTCGG |
| sgSLC25A1#1 Reverse | AAACCCGAGTACGTGAAGACGCAGC |
| sgSLC25A1#2 Forward | CACCGGAACTCGAACATTCCAAACC |
| sgSLC25A1#2 Reverse | AAACGGTTTGGAATGTTCGAGTTCC |
| sgSLC25A1#3 Forward | CACCGGACGGCTGGACAGCACGCGT |
| sgSLC25A1#3 Reverse | AAACACGCGTGCTGTCCAGCCGTCC |
| sgSLC25A1#4 Forward | CACCGGCACAATCTCCCTAACCCCG |
| sgSLC25A1#4 Reverse  qRT-PCR-GAPDH-Forward | AAACCGGGGTTAGGGAGATTGTGCC  AGCCACATCGCTCAGACAC |
| qRT-PCR-GAPDH-Reverse | GCCCAATACGACCAAATCC |
| qRT-PCR-SLC1A1-Forward | GGAGAAATTCTAATGCGGATGCTGA |
| qRT-PCR-SLC1A1-Reverse | TTCCGGATACGTTGGAATCCAGT |
| qRT-PCR-SLC1A2-Forward | GCCTGCCAACAGAGGACATCA |
| qRT-PCR-SLC1A2-Reverse | ACTATCCCAGCCCCAAAAGAGTCA |
| qRT-PCR-SLC1A3-Forward | ATGAGGCTTTGGCTGCCATTTT |
| qRT-PCR-SLC1A3- Reverse | AGGAATTCCAGCTGCCCCAATA |
| qRT-PCR-SLC1A6-Forward | AAACCTTGTGGAGGCCTGCTT |
| qRT-PCR-SLC1A6- Reverse | CTGGTTCCGTTCTCCACTGAGAAT |
| qRT-PCR-SLC1A7-Forward | ACTGCCCACCGATGACATCAC |
| qRT-PCR-SLC1A7- Reverse | ATATGGGCCATGATCCCCGCT |
| qRT-PCR-p21-Forward | CTTGTACCCTTGTGCCTCGCT |
| qRT-PCR-p21- Reverse | CGGCGTTTGGAGTGGTAGAAAT |
| qRT-PCR-HIST1H3D-Forward | AAGAAGCCCCACCGTTACCG |
| qRT-PCR-HIST1H3D- Reverse | AGTCTTGAAGTCCTGCGCGATCT |
| qRT-PCR-IL6-Forward | AACAAGCCAGAGCTGTGCAGAT |
| qRT-PCR-IL6- Reverse | AGCAGGCTGGCATTTGTGGTT |
| qRT-PCR-HIST1H2AC-Forward | CCAAAGCGAAATCCCGCTCTTCT |
| qRT-PCR-HIST1H2AC- Reverse | CGCTCTGCGTAGTTGCCTTTA |
| qRT-PCR-TP63-Forward | GTTGGGCTGTTCATCATGTCTGGA |
| qRT-PCR-TP63- Reverse | CCAGATCGCATGTCGAAATTGCTC |
| qRT-PCR-ATF3-Forward | CAGCTGCAAAGTGCCGAAACAA |
| qRT-PCR-ATF3- Reverse | CTCAATCTGAGCCTTCAGTTCAGC |
| qRT-PCR-GADD45A-Forward | TGCTCAGCAAAGCCCTGAGT |
| qRT-PCR-GADD45A- Reverse | AGGCAGGATCCTTCCATTGAGA |
| qRT-PCR-GDF15-Forward | CAGAGCTGGGAAGATTCGAACA |
| qRT-PCR-GDF15- Reverse | AGATACGCAGGTGCAGGTGG |
| qRT-PCR-DDIT3-Forward | TGCAAGAGGTCCTGTCTTCAGATG |
| qRT-PCR-DDIT3- Reverse | CAGAGAAGCAGGGTCAAGAGTGGT |
| qRT-PCR-XBP1-Forward | AGTTGTCACCCCTCCAGAACAT |
| qRT-PCR-XBP1- Reverse | TGGGTCCAAGTTGTCCAGAATGC |
| qRT-PCR-DNAJB9-Forward | CCCGGATGCTGAAGCAAAATTCAGAG |
| qRT-PCR-DNAJB9- Reverse | AGCACTGTGTCCAAGTGTATCATACTC |
| qRT-PCR-HIF0-Forward | TGTCCTCAAGCAGACCAAAGGG |
| qRT-PCR-HIF0- Reverse | TTGGCGTGGCTACCTTCTTGAT |
| qRT-PCR-GPR1-Forward | TCTGGTTGTGGTTGTGGCCTTT |
| qRT-PCR-GPR1- Reverse | ATTCCAGCCTGCATCACATGGT |
| qRT-PCR-PPP1R15A-Forward | CGCCCAGAAACCCCTACTCATGAT |
| qRT-PCR-PPP1R15A- Reverse | AGACAGCCAGGAAATGGACAGTGA |
| qRT-PCR-CTH-Forward | GCCCAGTTCCTGGAATCTAATCCTTG |
| qRT-PCR-CTH- Reverse | CTGTACACTGACGCTTCACCAACT |
| qRT-PCR-DMGDH-Forward | TCACCTTGGCAACGGATGATGT |
| qRT-PCR-DMGDH- Reverse | GCTGTAGCTATAGCTTCCAGATGTCG |
| qRT-PCR-VEGFA-Forward | CAATGACGAGGGCCTGGAGT |
| qRT-PCR-VEGFA- Reverse | TGTGCTGTAGGAAGCTCATCTCTC |
| qRT-PCR-TYMP-Forward | GACGGAATCCTATATGCAGCCAGAGA |
| qRT-PCR-TYMP- Reverse | ACAGCCCCTCCACGAGTTTCTTA |
| qRT-PCR-UPP1-Forward | ATGGAGTCCTCGGTGTTTGCC |
| qRT-PCR-UPP1- Reverse | ATTGCGAGGGCTGCTGATCT |
| qRT-PCR-ERRFI1-Forward | AACCATGGCCTACAGTCTGAACTC |
| qRT-PCR-ERRFI1- Reverse | TTCTGCAAAGCAGTGGCCATTC |
| qRT-PCR-HBEGF-Forward | ACAAGGACTTCTGCATCCATGGAG |
| qRT-PCR-HBEGF- Reverse | ATAAGCGATTTTCCACTGGGAGGC |
| qRT-PCR-SLC25A38-Forward | ATGGATTGGCCAAGCAGTGACA |
| qRT-PCR-SLC25A38- Reverse | CATTAGAGTTCTGCGGAGGGCT |
| qRT-PCR-CYP1A1-Forward | AAACCAGTGGCAGATCAACCATGACC |
| qRT-PCR-CYP1A1- Reverse | ACACCTTGTCGATAGCACCATCAG |
| qRT-PCR-KLHDC7B-Forward | AGCTCCAACTCCAGCCCCAA |
| qRT-PCR-KLHDC7B- Reverse | CTCCTGAGGCTTTGACCCGT |
| qRT-PCR-SLC22A15-Forward | CGACCTTCAGGTGTATTCGTATCGC |
| qRT-PCR-SLC22A15- Reverse | CTCACTCCCTAAAGAGCTCTCCTTGT |
| qRT-PCR-ASNS-Forward | TGGCGACCAAAAGAAGCCTTCA |
| qRT-PCR-ASNS- Reverse | GGCTGCATTTGCCATCATTGCATC |
|  |  |
|  |  |
